# Mortality burden and epidemiology of procedure- and device-related healthcare-associated infections in the United States, 1999–2023: a CDC WONDER analysis

**DOI:** 10.3389/fpubh.2025.1689828

**Published:** 2025-11-03

**Authors:** Lang Xie, Kaide Xia, Xiaodong Xu, Meisu Zhu, Hailing Li, Junwen Wang, Mei Chen

**Affiliations:** ^1^Department of Hospital Infection Management and Preventive Health Care, Zhejiang Provincial People's Hospital Bijie Hospital, Bijie, China; ^2^Guiyang Maternal and Child Health Care Hospital, Guiyang Children's Hospital, Guiyang, China; ^3^Department of Psychosomatic Medicine, The Second People’s Hospital of Guiyang, Guiyang, China; ^4^Department of Hospital Infection Management, The Third People’s Hospital of Bijie, Bijie, China

**Keywords:** procedure- and device-related infections, mortality trends, healthcare-associated infections, epidemiology, disparities in infection control, CDC WONDER database

## Abstract

**Background:**

Procedure- and device-related healthcare-associated infections (PD-HAIs) are a major cause of preventable hospital mortality, yet population-level data on long-term trends remain limited. This study aims to evaluate national PD-HAI mortality trends and subgroup disparities in the United States from 1999 to 2023.

**Methods:**

This descriptive study analyzed PD-HAI-related mortality in the United States from 1999 to 2023 using the CDC Wide-ranging Online Data for Epidemiologic Research (WONDER) Multiple Cause of Death database. Deaths involving PD-HAIs were identified using ICD-10 codes. Age-adjusted mortality rates (AAMRs) were calculated per 100,000 population using the 2000 U.S. standard population. Temporal trends were assessed using Joinpoint regression to estimate annual percent changes (APCs) and average annual percent changes (AAPCs) with 95% confidence intervals (CIs). Analyses were stratified by age, sex, race, region, urbanization, and state.

**Results:**

From 1999 to 2023, PD-HAI-related mortality declined markedly nationwide, with AAMRs falling from 1.62 to 0.77 per 100,000 and an overall AAPC of −3.02% (*p* < 0.05). The steepest declines occurred between 2001 and 2014. Reductions were observed across all demographic subgroups, although disparities persisted. Older adult individuals, males, Black populations, residents of the South, and nonmetropolitan areas consistently exhibited higher AAMRs. Black individuals experienced the greatest relative reduction (AAPC = −4.12%), while urban regions showed more pronounced declines than rural areas. State-level analyses revealed substantial heterogeneity in baseline mortality and trends. Although the overall infection-type distribution remained stable, deaths due to infections involving joint prostheses and other implants increased steadily over time.

**Conclusion:**

Despite national declines in PD-HAI mortality, trends have been less consistent since 2014, and significant geographic and demographic disparities persist. Sustained, state-tailored infection-prevention efforts—prioritizing jurisdictions with little or no progress—are needed as a matter of health equity to further reduce preventable deaths.

## 1 Introduction

Healthcare-associated infections (HAIs) pose a significant public health burden worldwide, contributing to elevated patient morbidity, mortality, and healthcare expenditures (1). Procedure- and device-related HAIs (PD-HAIs)—encompassing surgical site infections, cardiovascular device infections, prosthetic joint infections, catheter-associated urinary tract infections, and other implant-related infections—constitute a major proportion of preventable infectious complications and deaths in hospitalized populations (2, 3). The growing dependence on invasive procedures and implantable devices, particularly among older adult and multimorbid patients, has exacerbated the clinical and economic impact of PD-HAIs (4).

While infection prevention policies and device care standards in countries like the United States have reduced rates of specific infections, such as cardiac implant infections (5), and infections of electronic implantable devices (6), comprehensive population-level data on PD-HAI mortality remain scarce (7, 8). Systematic analyses of long-term mortality trends, particularly across demographic subgroups and geographic regions, are lacking (9). Additionally, the potential shifts in PD-HAI etiology following advances in clinical practice and preventive measures require further investigation (10, 11).

Against the backdrop of an aging U.S. population and increasing complexity of medical procedures, a systematic evaluation of long-term trends and distribution patterns of PD-HAI-related mortality holds significant practical implications. This study leverages the Centers for Disease Control and Prevention (CDC) Wide-ranging ONline Data for Epidemiologic Research (WONDER) database to analyze age-standardized mortality trends of PD-HAIs from 1999 to 2023, exploring heterogeneity in population structure and geographic distribution, as well as the evolving composition of different infection types. The findings aim to provide key evidence for optimizing infection prevention strategies, guiding healthcare resource allocation, and informing evidence-based public health policies.

## 2 Methods

### 2.1 Data source and population

This descriptive study analyzed mortality trends using death certificate data from the Wide-ranging ONline Data for Epidemiologic Research (WONDER) database maintained by the U.S. Centers for Disease Control and Prevention (CDC) (12). Records from the publicly available Multiple Cause of Death Data dataset were utilized, which encompasses comprehensive mortality data across all 50 U.S. States and the District of Columbia (13, 14). Procedure- and device-related healthcare-associated infections (PD-HAIs)-associated deaths were identified based on International Classification of Diseases, Tenth Revision (ICD-10) codes. Cases were included for analysis if PD-HAIs-related codes were documented on the death certificate (detailed codes are provided in Supplementary Table 1). The primary objective of this study was to assess the burden and temporal trends of PD-HAIs-associated mortality in the United States from 1999 to 2023. As this study utilized publicly available, anonymized federal data, institutional review board approval was not required. The reporting of this study followed the Strengthening the Reporting of Observational Studies in Epidemiology (STROBE) guidelines.

### 2.2 Data processing

Demographic and mortality-related variables (e.g., age, sex, census region, urbanization level) remained consistent between the 1999–2020 and 2018–2023 CDC WONDER datasets, except the race variable. To ensure comparability across periods with differing race classification methods (bridged race classification for the earlier period versus single-race classification for the later period), the race variable was standardized into three categories: White people, Black, and Other. Age was dichotomized into non-older adult (<65 years) and older adult (≥65 years). Urbanization status was consolidated based on the 2013 U.S. Census data: counties with populations <50,000 were classified as nonmetropolitan, while others were designated as metropolitan. Geographic regions were categorized according to U.S. Census Bureau standards (Northeast, Midwest, South, and West) (15).

### 2.3 Statistical analysis

Age-adjusted mortality rates (AAMRs) per 100,000 population were calculated to evaluate national PD-HAIs-related mortality trends, standardized to the 2000 U.S. population (16). Temporal changes in mortality were analyzed using the Joinpoint Regression Program, which employs a log-linear regression model to estimate annual percentage changes (APCs) with corresponding 95% confidence intervals (CIs) (17). We did not force any *a priori* joinpoints; they were selected by permutation tests. The program also computed average annual percentage changes (AAPCs) to assess overall trends across the study period. Trends were classified as increasing or decreasing based on deviations from the null hypothesis (no significant trend). Statistical significance was set at *p* < 0.05 (two-tailed t-test).

## 3 Results

### 3.1 National Trends in PD-HAIs–related mortality

In 1999, the United States recorded 4,406 PD-HAI-related deaths, with an AAMR of 1.62 per 100,000 (95% CI: 1.58–1.67); by 2023, deaths declined to 3,299, accompanied by a reduction in AAMR to 0.77 (95% CI: 0.74–0.79). The average annual percent change (AAPC) of −3.02% (95% CI: −3.55 to −2.52) reflects a statistically significant decline in mortality over the study period (Table 1). Joinpoint regression revealed three distinct trends: a nonsignificant AAMR increase from 1999 to 2001 (APC = 3.46%, *p* = 0.42), followed by a pronounced decrease between 2001 and 2014 (APC = −6.21%, *p* = 0.021), and stabilization from 2014 to 2023 (APC = 0.32%, *p* = 0.61) (Supplementary Figure 1).

**Table 1:** Mortality burden of procedure- and device-related HAIs in the United States, 1999–2023.

Characteristics	1999		2023		AAPC
	Counts	AAMR (95% CI)	Counts	AAMR (95% CI)	
Total	4406	1.62 (1.58, 1.67)	3299	0.77 (0.74, 0.79)	−3.02 (−3.55 to −2.52)
Age					
Non-older adult	1251	0.54 (0.51, 0.57)	858	0.24 (0.23, 0.26)	−3.37 (−4.2 to −2.68)
Older adult	3155	9.12 (8.80, 9.44)	2441	4.37 (4.20, 4.55)	−3.09 (−3.54 to −2.64)
Census region					
Northeast	794	1.39 (1.29, 1.49)	569	0.73 (0.67, 0.79)	−3.12 (−3.83 to −2.28)
Midwest	1063	1.63 (1.53, 1.73)	713	0.80 (0.74, 0.86)	−2.89 (−3.35 to −2.44)
South	1782	1.84 (1.76, 1.93)	1250	0.75 (0.71, 0.79)	−3.53 (−3.97 to −3.08)
West	767	1.41 (1.31, 1.51)	767	0.80 (0.75, 0.86)	−2.28 (−3.13 to −1.7)
Sex					
Female	2250	1.43 (1.37, 1.49)	1418	0.60 (0.57, 0.63)	−3.25 (−3.73 to −2.72)
Male	2156	1.90 (1.82, 1.99)	1881	1.00 (0.95, 1.05)	−2.76 (−3.33 to −2.23)
Race					
Black	771	3.10 (2.88, 3.32)	487	1.05 (0.95, 1.14)	−4.12 (−4.67 to −3.58)
Other	92	1.13 (0.89, 1.40)	151	0.45 (0.37, 0.52)	−3.68 (−4.86 to −2.81)
White people	3543	1.47 (1.42, 1.52)	2661	0.78 (0.75, 0.81)	−2.66 (−3.17 to −2.16)
Urbanization					
Metropolitan	3495	1.57 (1.52, 1.63)	2383^a^	0.69 (0.66, 0.71)	−4.05 (−4.41 to −3.68)
Nonmetropolitan	911	1.81 (1.69, 1.92)	569^a^	0.88 (0.81, 0.96)	−3.21 (−3.82 to −2.55)

^a^Indicates data for the year 2020, as CDC WONDER did not provide data for this category from 2021 to 2023.

### 3.2 Trends in PD-HAIs–related mortality by age

**Figure 1:**
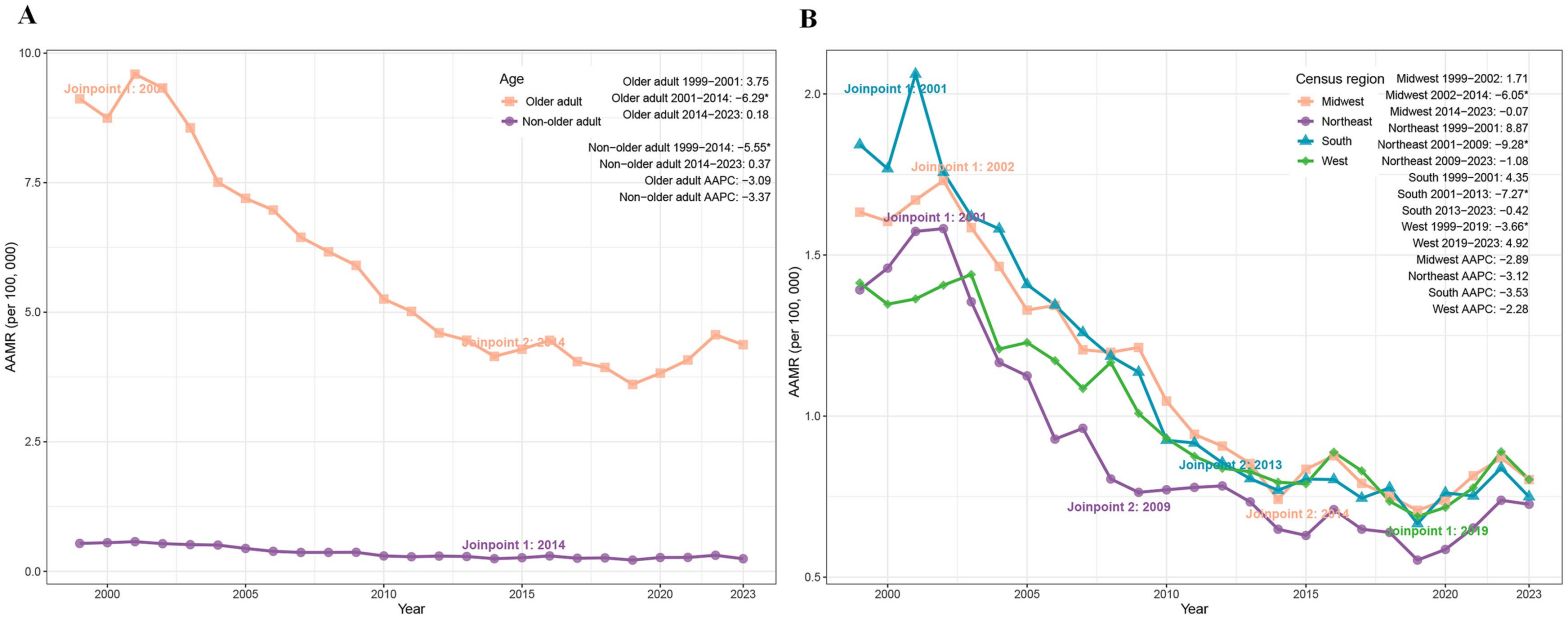
Trends of AAMR in PD-HAI-related mortality by age **(A)** and census region **(B)** in the United States (1999–2023). PD-HAI: procedure- and device-related healthcare-associated infections, AAMR, age-adjusted mortality rates.

Between 1999 and 2023, PD-HAI-associated AAMR declined markedly in both non-older adult and older adult populations. Non-older adult deaths fell from 1,251 to 858, accompanied by an AAMR reduction from 0.54 to 0.24 (AAPC = −3.37%; *p* < 0.05), while older adult deaths decreased from 3,155 to 2,441 with a corresponding AAMR decline from 9.12 to 4.37 (AAPC = −3.09%; *p* < 0.05) (Table 1). Joinpoint regression revealed a significant AAMR reduction in non-older adult individuals during 1999–2014 (APC = −5.55%; p < 0.05), followed by stabilization. Among older adult patients, the AAMR showed a pronounced decrease from 2001 to 2014 (APC = −6.29%; *p* = 0.004), with no significant trends in other intervals (Figure 1A).

### 3.3 Trends in PD-HAIs–related mortality by census region

The age-adjusted mortality rate (AAMR) for PD-HAIs declined across all four major U.S. census regions between 1999 and 2023. While the South maintained the highest mortality burden, its AAMR dropped from 1.84 to 0.75 (AAPC = −3.53%), followed by the Northeast (1.39 to 0.73; AAPC = −3.12%), Midwest (1.63 to 0.80; AAPC = −2.89%), and West (1.41 to 0.80; AAPC = −2.28%) (Table 1). Joinpoint regression identified distinct temporal patterns: the Midwest exhibited a sharp decline from 2002 to 2014 (APC = −6.05%, *p* < 0.01), while the Northeast showed rapid reduction between 2001 and 2009 (APC = −9.28%, *p* = 0.02). The South demonstrated significant decreases from 2001 to 2013 (APC = −7.27%, *p* = 0.002), whereas the West’s decline from 1999 to 2019 (APC = −3.66%, *p* = 0.007) was followed by a non-significant increase through 2023 (Figure 1B).

### 3.4 Trends in PD-HAIs–related mortality by sex

**Figure 2:**
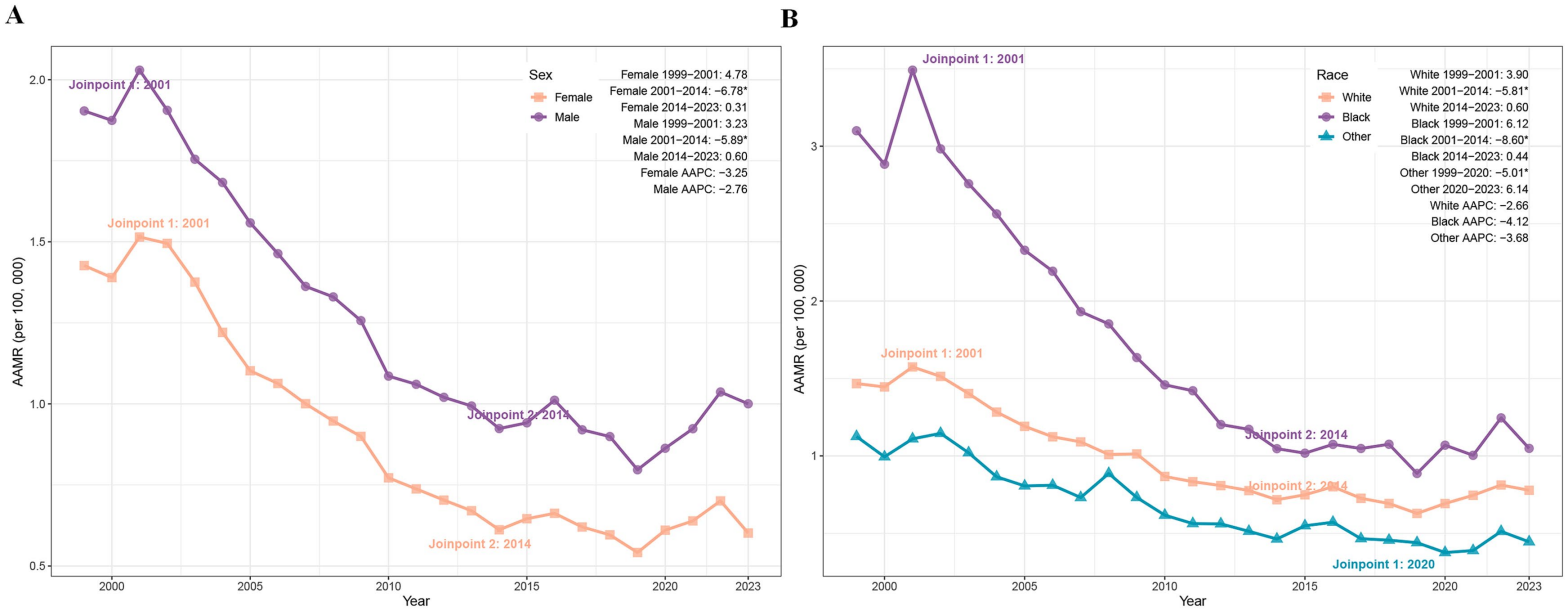
Trends of AAMR in PD-HAI-related mortality by sex **(A)** and race **(B)** in the United States (1999–2023). PD-HAI, procedure- and device-related healthcare-associated infections, AAMR, age-adjusted mortality rates.

Between 1999 and 2023, the AAMR for PD-HAIs declined significantly in both sexes. Female deaths fell from 2,250 to 1,418, accompanied by an AAMR reduction from 1.43 to 0.60 (AAPC = −3.25%, *p* < 0.05), while male deaths decreased from 2,156 to 1,881, with AAMR declining from 1.90 to 1.00 (AAPC = −2.76%, *p* < 0.05) (Table 1). Joinpoint analysis revealed a pronounced AAMR decrease among females from 2001 to 2014 (APC = −6.78%, *p* = 0.003), with stable trends thereafter. Similarly, males exhibited a significant AAMR reduction during 2001–2014 (APC = −5.89%, *p* = 0.039), followed by periods of nonsignificant change (Figure 2A).

### 3.5 Trends in PD-HAIs–related mortality by race

The AAMR for PD-HAIs-related mortality declined significantly in all racial groups. Black individuals experienced a reduction in deaths from 771 to 487, with the AAMR falling from 3.10 to 1.05 (AAPC = −4.12%, *p* < 0.001), while White people deaths decreased from 3,543 to 2,661, accompanied by an AAMR decline from 1.47 to 0.78 (AAPC = −2.66%, *p* = 0.004). In other racial groups, deaths rose from 92 to 151 despite an AAMR decrease from 1.13 to 0.45 (AAPC = −3.68%, *p* = 0.007) (Table 1). Joinpoint regression revealed a pronounced AAMR decline among Black individuals between 2001 and 2014 (APC = −8.60%, p < 0.001), paralleled by a significant reduction among White peoples during the same period (APC = −5.81%, *p* = 0.019). Other races exhibited a sustained decline from 1999 to 2020 (APC = −5.01%, *p* = 0.036), followed by a marginal post-2020 increase that lacked statistical significance (Figure 2B).

### 3.6 Trends in PD-HAIs–related mortality by urbanization

**Figure 3:**
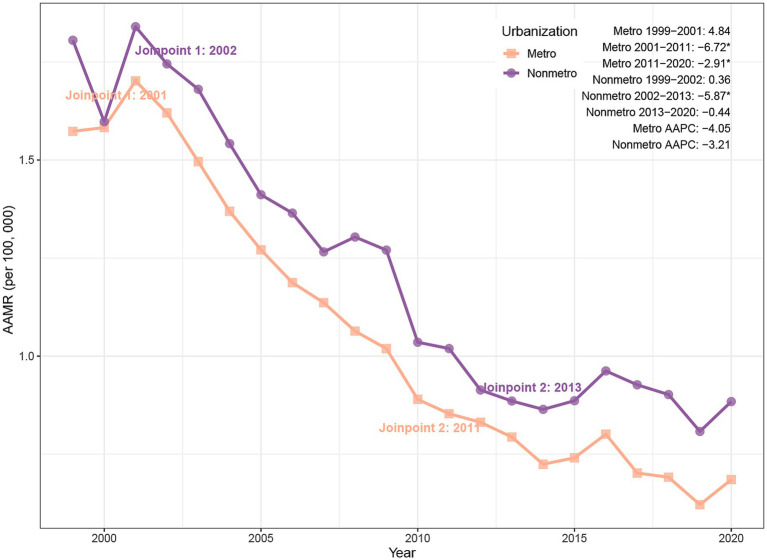
Trends of AAMR in PD-HAI-related mortality by urbanization in the United States (1999–2020). PD-HAI, procedure- and device-related healthcare-associated infections, AAMR, age-adjusted mortality rates.

Between 1999 and 2020, PD-HAIs-related mortality exhibited a significant decline in AAMR across both urban and non-urban regions. Urban areas recorded a reduction in deaths from 3,495 to 2,383, with the AAMR falling from 1.57 to 0.69 (AAPC = −4.05%, *p* < 0.001). Non-urban areas similarly showed a decrease from 911 to 569 deaths, accompanied by an AAMR decline from 1.81 to 0.88 (AAPC = −3.21%, *p* = 0.002) (Table 1). Joinpoint analysis revealed a pronounced AAMR reduction in urban settings between 2001 and 2011 (APC = −6.72%, p < 0.001), followed by a slower yet still significant decrease from 2011 to 2020 (APC = −2.91%, *p* = 0.005). In non-urban areas, the AAMR declined markedly from 2002 to 2013 (APC = −5.87%, *p* = 0.016), while remaining stable during other intervals (Figure 3).

### 3.7 Trends in PD-HAIs–related mortality by states

**Figure 4:**
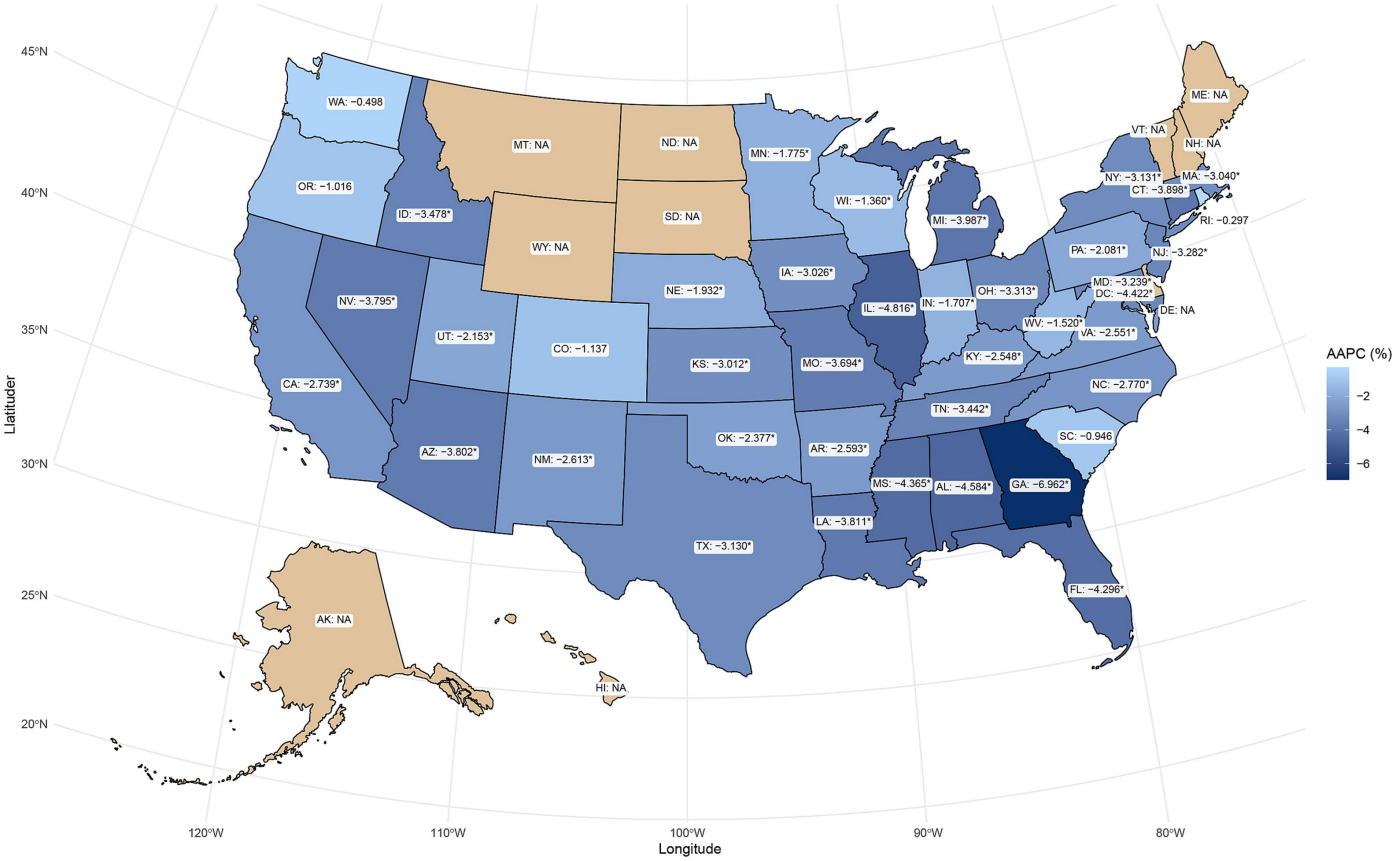
Geographic distribution of AAPC in PD-HAI-related mortality by state in the United States. PD-HAI, procedure- and device-related healthcare-associated infections, AAPC, average annual percent change.

From 1999 to 2023, the AAMR for PD-HAIs-related mortality showed a significant decline in most states, but there were significant differences in baseline levels and the magnitude of changes. The five states with the highest AAMR in 1999 were District of Columbia (5.05 per 100,000), New Mexico (2.76), Mississippi (2.73), Georgia (2.78), and Connecticut (2.51). By the end of the study period, the five states with the highest AAMR were the District of Columbia (3.49 in 2008), New Mexico (1.48), Washington (1.15), Mississippi (1.03), and Rhode Island (2.07 in 2002). When ranked by AAPC, the five states with the largest decreases were Georgia (−6.96%), Illinois (−4.82%), Mississippi (−4.37%), Alabama (−4.58%), and Florida (−4.30%), showing a significant downward trend. Conversely, changes in Washington, Colorado, Oregon, South Carolina, and Rhode Island were not statistically significant (Supplementary Table 2; Figure 4). Taken together, several jurisdictions showed limited or non-significant improvement post-2014, indicating the need for targeted, state-specific support to advance equity.

### 3.8 Distribution of Infection types among PD-HAIs–related deaths

**Figure 5:**
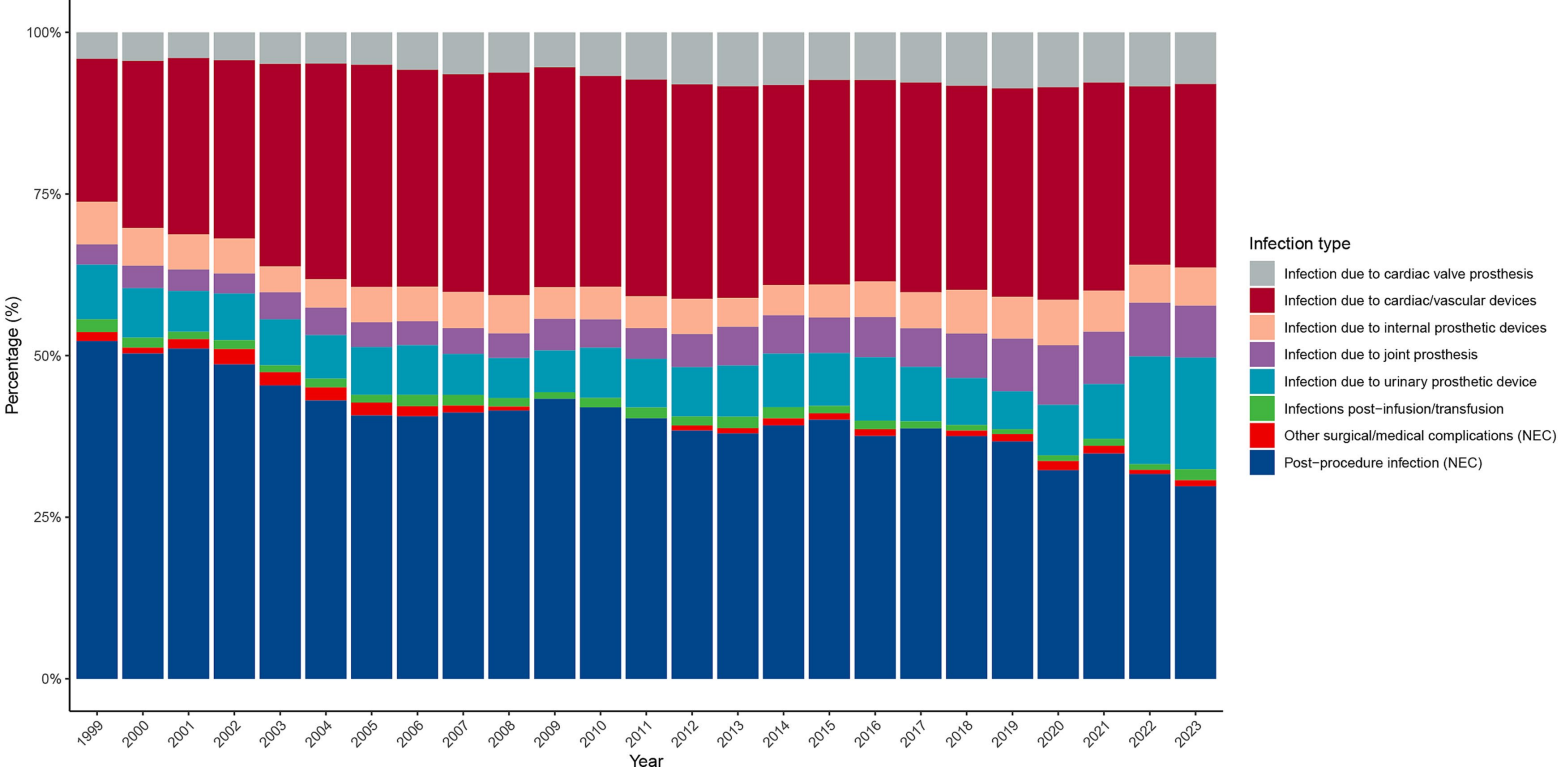
Distribution of PD-HAI-related mortality by infection type in the United States (1999–2023). PD-HAI, procedure- and device-related healthcare-associated infections.

Between 1999 and 2023, the distribution of infection types contributing to PD-HAI-related mortality changed substantially (Figure 5). Postoperative infections and cardiac/vascular device-related infections predominated throughout this period, representing over 50 and 30% of cases in 1999, respectively, and maintaining their leading positions in 2023 at approximately 30 and 25%. Since 2005, joint prosthesis- and implanted device-associated infections have risen steadily, collectively surpassing 15% by 2023. While infusion/transfusion-related and urinary system prosthesis infections have consistently represented minor proportions, both categories exhibited modest growth in later years. Conversely, cardiac valve prosthesis infections and those linked to other surgical or medical complications have persistently accounted for less than 10% of cases, with negligible variation. Although the overall pattern of infection types has remained largely consistent, device-related infections have progressively gained prominence over the study period.

## 4 Discussion

This study systematically evaluated mortality trends associated with PD-HAIs using U.S. national death registration data from 1999 to 2023. The analysis revealed a substantial decline in PD-HAI-related mortality over the past two decades, particularly between 2001 and 2014, coinciding with major advancements in infection control and healthcare quality. Despite this progress, persistent disparities emerged across age groups, genders, racial demographics, geographic regions, and urbanization levels, highlighting ongoing challenges in equitable healthcare delivery. The attenuation and heterogeneity of trends observed since 2014 likely result from several factors, including diminishing returns following earlier national initiatives, shifts in case-mix and device complexity, and evolving antimicrobial resistance. Changes in surveillance definitions and reporting incentives may also have tempered the measured declines. Supporting this interpretation, state-level differences in baseline burden, resources, reporting practices, and implementation fidelity indicate that sustaining improvements will demand ongoing and contextually adaptive quality-improvement efforts.

From the overall trend, the AAPC of the AAMR for PD-HAIs showed a significant decline, consistent with previous studies, reflecting continuous improvements in infection control (18, 19). This decline was particularly pronounced between 2001 and 2014 and may be linked to several hospital infection control policies introduced by the federal government in the early 2000s, such as the implementation of the Hospital Infection Reporting System (HAI Reporting), which encouraged hospitals to enhance infection prevention measures (20, 21). Additionally, the adoption of national policies such as “zero tolerance for infections” accelerated systematic improvements in hospitals regarding aseptic techniques, antimicrobial stewardship, and patient safety (22, 23). Collectively, these policies contributed to the significant decline in healthcare-associated infections and their related mortality rates.

In age-stratified analyses, although the mortality rate was higher in the older adult population compared to the non-older adult population, both groups showed a decreasing trend, indicating that infection control measures have benefited diverse populations (19, 24). However, the older adult continue to be a priority target for PD-HAI prevention due to impaired immune function, underlying comorbidities, and higher hospitalization rates (25). Regarding gender differences, the age-adjusted mortality rate (AAMR) for males remained higher than that for females throughout the study period, with a relatively smaller decline, potentially related to higher rates of severe illness, surgical procedures, or comorbidities among males during hospitalization (26, 27). Similar trends were observed in racial subgroup analyses. Although the Black population had the highest absolute mortality rate, it also experienced the most significant decline, suggesting that interventions targeting high-risk populations may have achieved some success (28). Nevertheless, racial disparities remain significant, underscoring the need for further investigation into the impacts of socioeconomic status, healthcare accessibility, and structural inequities on PD-HAI outcomes (29–31).

From a regional perspective, the southern United States has consistently experienced the highest PD-HAI mortality burden, which is characterized by relatively limited healthcare resources and a high burden of chronic diseases (32). In contrast, while the Midwest and Northeast initially had slightly lower mortality rates, they experienced a rapid decline in the mid-2000s, suggesting that regional interventions targeting hospital infections and investments in public health resources may have played a key role (33). Notably, the Western region saw a slight increase in mortality rates after 2019, though this was not statistically significant. Nonetheless, ongoing attention should be given to potential impacts of changes in healthcare policies, demographic shifts, or fluctuations in data quality on these trends (34). Urbanization levels also showed significant differences. Urban areas exhibited relatively lower age-adjusted mortality rates (AAMRs) and greater declines, which may be attributed to more advanced medical facilities, well-established infection prevention systems, and higher accessibility to healthcare services (35, 36). However, PD-HAI-related mortality rates in non-urban areas remain consistently high, highlighting the ongoing challenge posed by uneven distribution of medical resources between urban and rural areas for infection control. Strengthening infection management capacity in primary healthcare institutions and expanding public health service coverage is necessary (37). Beyond urban–rural contrasts, system-capacity indicators—particularly infection-preventionist (IP) staffing—are associated with HAI performance; recent multi-center studies report median staffing near 1 IP per ~120–170 beds and higher HAI rates with understaffing (38). Public health funding also varies widely by state. The post-2019 increase in the West likely reflects within-region heterogeneity (state/hospital level), pandemic-era care disruptions, and case-mix shifts rather than resource abundance per se (39, 40). Across strata, PD-HAI AAMRs showed increases between 2020 and 2023. These fluctuations are consistent with contemporaneous NHSN reports of national HAI increases during the COVID-19 period; however, given known death-certificate coding limitations and pandemic-related care-delivery disruptions, we refrain from causal attribution. These observations underscore the need to strengthen hospital infection surveillance and emergency preparedness (39, 41).

Further analysis revealed that the composition of infection types associated with PD-HAI-related deaths has remained relatively stable, with postoperative infections and infections related to cardiovascular devices dominating (19, 42). With advancements in medical technology and the increasing use of implantable devices such as artificial joints, the proportion of infections associated with joint prostheses and other implantable devices has steadily increased, a trend reflected in multiple studies (43, 44). This suggests that future infection control efforts should prioritize the sterile management of high-risk medical devices, strict adherence to surgical procedures, and improvements in the quality of postoperative care (45). Additionally, early diagnosis and preventive measures for implant-related infections are crucial to reducing associated mortality rates (46, 47).

This study analyzed standardized mortality data spanning over 20 years across the United States, employing Joinpoint regression to quantify trends in PD-HAI-related mortality, which provided robust population-level insights. Several limitations should be acknowledged. First, the CDC WONDER database relies on death certificates, where coding inconsistencies and underreporting may occur due to variations in documentation practices, potentially compromising data accuracy. Second, PD-HAI identification through ICD-10 codes might miss complex or ambiguously documented infections, introducing possible misclassification. Third, incomplete subgroup data (e.g., by race or state) for certain years reduced the precision of trend estimates and generalizability for specific populations. Furthermore, the aggregated nature of these data precluded adjustments for clinical, healthcare system, or socioeconomic confounders, limiting causal interpretation. State-level differences in surveillance and implementation may also contribute to residual variation, reinforcing the need for tailored, equity-oriented approaches. Future research should incorporate individual-level clinical data to better characterize infection determinants and refine intervention strategies.

In conclusion, despite significant declines in PD-HAI-related mortality nationwide, older adults, certain racial groups, and residents of southern and non-urban areas continue to experience disproportionately high mortality rates with slower improvements. While mortality declined substantially—especially from 2001 to 2014—post-2014 trajectories were less consistent. These persistent disparities underscore the urgency of tailored infection control measures, particularly in primary care and underserved regions. Concurrently, evolving patterns of device-associated infections necessitate stricter perioperative protocols, sustained surveillance, and innovations in antimicrobial materials to further mitigate PD-HAI risks.

## Data Availability

The datasets presented in this study can be found in online repositories (https://wonder.cdc.gov/). The names of the repository/repositories and accession number(s) can be found in the article/Supplementary material.
