# Supplementary material for: Mortality burden and epidemiology of procedure- and device-related healthcare-associated infections in the United States, 1999–2023: a CDC WONDER analysis
Source: Front Public Health. 2025 Nov 3;13:1689828. doi: 10.3389/fpubh.2025.1689828 (PMC12620361; doi:10.3389/fpubh.2025.1689828)
Supplement: Supplementary file 1 [file Data_Sheet_1.PDF]

Supplementary appendix

Supplementary to:

Mortality Burden and Epidemiology of Procedure- and Device-Related Healthcare-Associated Infections in the United States, 1999–2023: A CDC WONDER Analysis

Lang Xie[1], Kaide Xia[2], Xiaodong Xu[1], Meisu Zhu[1], Hailing Li[1], Junwen Wang[3*], Mei Chen[4*]

Contents

Supplementary figures: 3

Supplementary Figure 1. Trends of AAMR in PD-HAI-Related Mortality in the United States (1999–2023). 3

Supplementary tables: 4

Supplementary Table 1. ICD-10 codes and corresponding infection types included in this study 4

Supplement Table 2. Mortality Burden of Procedure- and Device-Related HAIs in the United States by States 6

Supplementary figures:

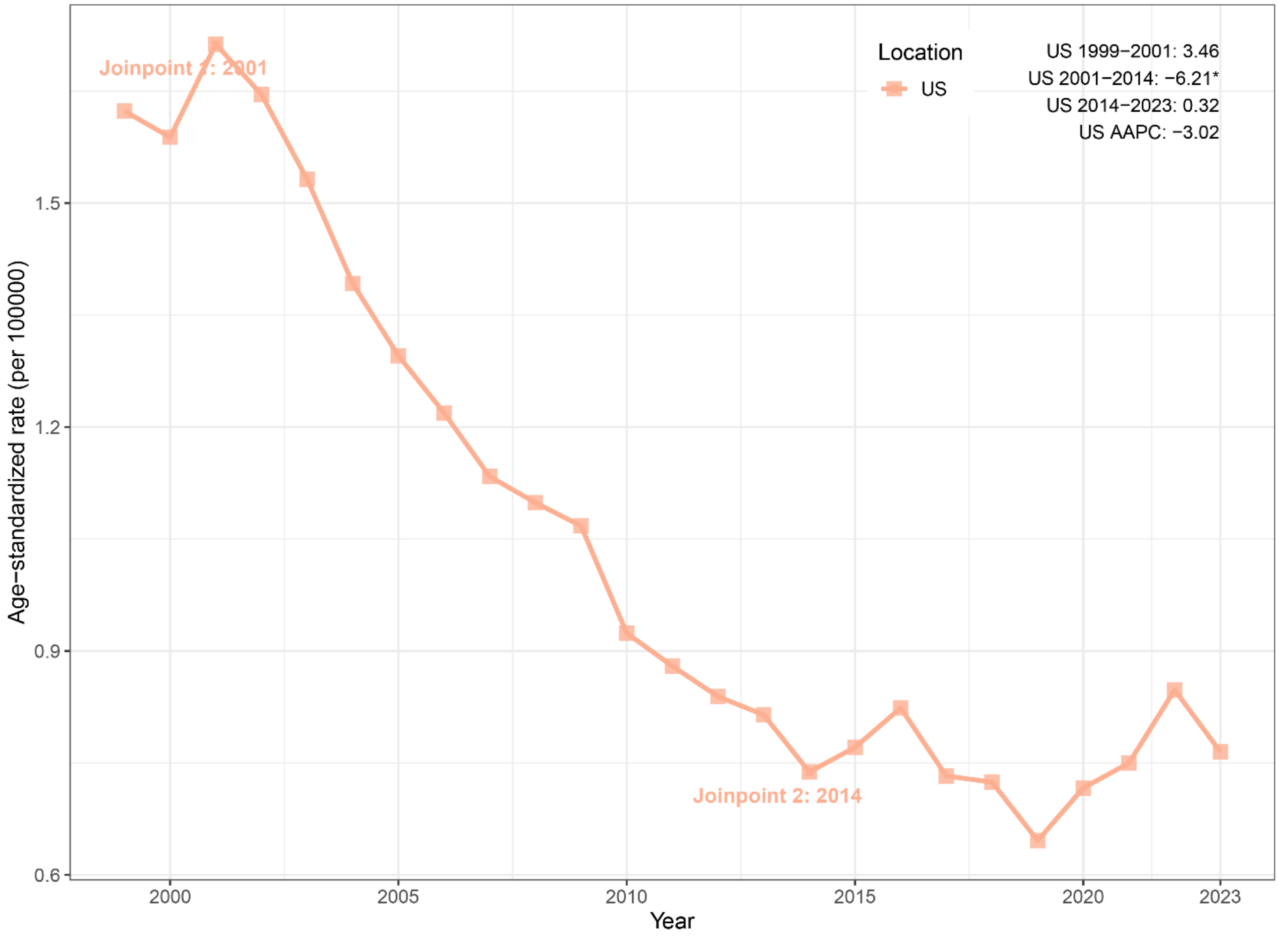

Supplementary Figure 1. Trends of AAMR in PD-HAI-Related Mortality in the United States (1999–2023).

PD-HAI: procedure- and device-related healthcare-associated infections, AAMR: age-adjusted mortality rates

Supplementary tables:

Supplementary Table 1. ICD-10 codes and corresponding infection types included in this study

ICD-10	Infection type	Infection Type (Abbreviation)
T80.2	Infections following infusion, transfusion and therapeutic injection	Infections post-infusion/transfusion
T81.4	Infection following a procedure, not elsewhere classified	Post-procedure infection (NEC)
T82.6	Infection and inflammatory reaction due to cardiac valve prosthesis	Infection due to cardiac valve prosthesis
T82.7	Infection and inflammatory reaction due to other cardiac and vascular devices, implants and grafts	Infection due to cardiac/vascular devices
T83.5	Infection and inflammatory reaction due to prosthetic device, implant and graft in urinary system	Infection due to urinary prosthetic device
T84.5	Infection and inflammatory reaction due to internal joint prosthesis	Infection due to joint prosthesis
T85.7	Infection and inflammatory reaction due to other internal prosthetic devices, implants and grafts	Infection due to internal prosthetic devices
T88.8	Other specified complications of surgical and medical care, not elsewhere classified	Other surgical/medical complications (NEC)

Supplement Table 2. Mortality Burden of Procedure- and Device-Related HAIs in the United States by States

Location	Initial			Final			AAPC (95% CI)
	Year	Counts	AAMR (95% CI)	Year	Counts	AAMR (95% CI)	
Alabama	1999	85	1.9 (1.52,2.35)	2023	46	0.76 (0.55,1.02)	-4.58 (-6.62 to -2.86)
Arizona	1999	78	1.53 (1.21,1.9)	2023	65	0.64 (0.49,0.83)	-3.8 (-4.78 to -2.85)
Arkansas	1999	60	2.09 (1.59,2.69)	2023	34	0.87 (0.6,1.23)	-2.59 (-3.95 to -1.39)
California	1999	369	1.27 (1.14,1.4)	2023	292	0.63 (0.56,0.7)	-2.74 (-3.35 to -2.09)
Colorado	1999	52	1.52 (1.13,2)	2023	68	1.03 (0.8,1.31)	-1.14 (-2.69 to 0.59)
Connecticut	1999	93	2.51 (2.03,3.08)	2023	44	0.9 (0.65,1.22)	-3.9 (-4.74 to -3.02)
District of Columbia	1999	28	5.05 (3.35,7.3)	2008	20	3.49 (2.13,5.39)	-4.42 (-7.14 to -2.12)
Florida	1999	343	1.74 (1.56,1.93)	2023	203	0.58 (0.5,0.67)	-4.3 (-5.02 to -3.48)
Georgia	1999	182	2.78 (2.37,3.19)	2023	74	0.62 (0.48,0.78)	-6.96 (-8.15 to -5.78)
Idaho	1999	23	2 (1.27,3)	2022	23	1.02 (0.64,1.54)	-3.48 (-4.46 to -2.46)
Illinois	1999	258	2.17 (1.91,2.44)	2023	102	0.66 (0.53,0.8)	-4.82 (-5.73 to -4.09)
Indiana	1999	87	1.46 (1.17,1.8)	2023	80	0.96 (0.76,1.2)	-1.71 (-2.74 to -0.58)

Iowa	1999	68	1.99 (1.54,2.52)	2023	47	1.09 (0.8,1.45)	-3.03 (-4.6 to -1.62)
Kansas	1999	31	1.1 (0.75,1.56)	2023	27	0.78 (0.51,1.14)	-3.01 (-5.07 to -1.27)
Kentucky	1999	54	1.4 (1.05,1.82)	2023	54	0.96 (0.72,1.26)	-2.55 (-3.74 to -1.27)
Louisiana	1999	74	1.81 (1.42,2.27)	2023	35	0.57 (0.4,0.8)	-3.81 (-5.04 to -2.27)
Maryland	1999	103	2.15 (1.73,2.56)	2023	81	1.05 (0.83,1.31)	-3.24 (-5.06 to -2.03)
Massachusetts	1999	44	0.67 (0.48,0.89)	2023	52	0.57 (0.42,0.75)	-3.04 (-4.7 to -1.53)
Michigan	1999	141	1.47 (1.23,1.72)	2023	85	0.63 (0.5,0.79)	-3.99 (-5.38 to -2.85)
Minnesota	1999	62	1.28 (0.98,1.64)	2023	65	0.88 (0.68,1.13)	-1.78 (-2.85 to -0.69)
Mississippi	1999	74	2.73 (2.14,3.42)	2023	35	1.03 (0.71,1.46)	-4.37 (-6.22 to -3.06)
Missouri	1999	90	1.52 (1.23,1.87)	2023	52	0.63 (0.47,0.83)	-3.69 (-5.88 to -2.61)
Nebraska	1999	27	1.45 (0.95,2.1)	2023	24	1.07 (0.68,1.6)	-1.93 (-3.43 to -0.48)
Nevada	1999	31	1.79 (1.21,2.55)	2023	31	0.81 (0.54,1.16)	-3.79 (-5.09 to -1.65)
New Jersey	1999	140	1.61 (1.34,1.88)	2023	85	0.7 (0.56,0.87)	-3.28 (-4.48 to -2.21)
New Mexico	1999	45	2.76 (2.01,3.69)	2023	42	1.48 (1.05,2.01)	-2.61 (-4.84 to -1.34)
New York	1999	227	1.17 (1.02,1.32)	2023	177	0.66 (0.56,0.75)	-3.13 (-4.07 to -1.8)

North Carolina	1999	133	1.77 (1.47,2.07)	2023	124	0.92 (0.76,1.09)	-2.77 (-3.95 to -1.65)
Ohio	1999	219	1.88 (1.63,2.13)	2023	128	0.83 (0.68,0.98)	-3.31 (-4.4 to -2.25)
Oklahoma	1999	48	1.37 (1.01,1.81)	2023	47	0.98 (0.72,1.31)	-2.38 (-3.87 to -0.97)
Oregon	1999	41	1.18 (0.85,1.6)	2023	65	1.17 (0.89,1.49)	-1.02 (-2.82 to 0.85)
Pennsylvania	1999	222	1.54 (1.34,1.75)	2023	149	0.81 (0.67,0.94)	-2.08 (-3.25 to -0.71)
Rhode Island	2000	24	2.08 (1.33,3.09)	2002	25	2.07 (1.34,3.06)	-0.3 (-14.21 to 16.22)
South Carolina	1999	59	1.51 (1.15,1.95)	2023	71	0.99 (0.77,1.26)	-0.95 (-2.17 to 0.79)
Tennessee	1999	138	2.49 (2.07,2.91)	2023	92	1.05 (0.84,1.29)	-3.44 (-4.68 to -2.62)
Texas	1999	284	1.70 (1.50,1.90)	2023	231	0.74 (0.64,0.84)	-3.13 (-4.51 to -2.03)
Utah	1999	25	1.63 (1.06,2.41)	2023	30	0.97 (0.65,1.4)	-2.15 (-3.66 to -0.7)
Virginia	1999	78	1.24 (0.98,1.55)	2023	86	0.79 (0.62,0.98)	-2.55 (-4.03 to -1.2)
Washington	1999	72	1.34 (1.05,1.69)	2023	106	1.15 (0.93,1.37)	-0.5 (-1.73 to 0.97)
West Virginia	1999	23	1.08 (0.69,1.63)	2023	22	0.86 (0.53,1.31)	-1.52 (-2.98 to -0.12)
Wisconsin	1999	70	1.26 (0.98,1.59)	2023	82	1.01 (0.8,1.26)	-1.36 (-2.79 to -0.02)
